# Visual brokerage: Communicating data and research through visualisation

**DOI:** 10.1177/0963662518756853

**Published:** 2018-02-05

**Authors:** William L. Allen

**Affiliations:** University of Oxford, UK

**Keywords:** data visualisation, knowledge brokerage, migration, public engagement, research communication

## Abstract

Researchers increasingly use visualisation to make sense of their data and communicate findings more widely. But these are not necessarily straightforward processes. Theories of knowledge brokerage show how sociopolitical contexts and intermediary organisations that translate research for public audiences shape how users engage with evidence. Applying these ideas to data visualisation, I argue that several kinds of brokers (such as data collectors, designers and intermediaries) link researchers and audiences, contributing to the ways that people engage with visualisations. To do this, I draw on qualitative focus groups that elicited non-academic viewers’ reactions to visualisations of data about UK migration. The results reveal two important features of engagement: perceptions of brokers’ credibility and feelings of surprise arising from visualisations’ content and design. I conclude by arguing that researchers, knowledge brokers and the public produce – as well as operate within – a complex visualisation space characterised by mutual, bi-directional connections.

Researchers increasingly produce and have access to more data of different types. But communicating findings from these datasets is not always easy. Their sizes can make detecting patterns in conventional lists or tables difficult. Some datasets have numerous variables or are missing elements. These issues, applicable to ‘small’ as well as ‘big’ datasets (Gray et al., 2015), can make them complicated to understand even for experts who regularly use them.

To overcome these challenges, many researchers use visualisations to analyse and communicate their data and findings. Data visualisation is ‘the representation and presentation of data to facilitate understanding’ (Kirk, 2016: 19). Examples include charts of various types (e.g. bar, line and scatterplots) and maps that can include static or interactive features, such as search bars and the ability to reveal extra information by hovering over an image. Visualisations serve dual roles, sometimes simultaneously. They can explain trends, outliers or pre-selected key points, as well as enable users to explore data according to their own interests (Welles and Meirelles, 2015). As free and low-cost visualisation tools become more widespread and audiences increasingly encounter data and research visually, presenting research in interactive ways is clearly appealing.

Of course, visual representations of scientific data and ideas are not new: ‘the visual has historically been central to science communication’ (Bucchi and Saracino, 2016: 813). Pioneers of visualisation, notably Tufte (1983), advocated for designs that prioritised the data over other elements. This preference for simplicity, as a way of helping viewers make sense of an image, appears in guidance for communicating science through other media such as photography (Frankel, 2002). Building on this legacy, scholars have recently turned their attention to document the complex ways that users react to and engage with visualisations. Since visualisations communicate messages or viewpoints through their graphical elements (Kennedy et al., 2016a; Locke, 2011; Welles and Meirelles, 2015), they can impact users’ perceptions or generate emotional responses (Bucchi and Saracino, 2016; Herring et al., 2017; Kennedy and Hill, 2017). Moreover, the fact that they are often ‘re-presented in different contexts that invest them with different meanings’ (Locke, 2011: 304) further complicates their status as objects that are ‘multifaceted and “multitruthed”’ (Welles and Meirelles, 2015: 37).

Given these qualities of visualisations, there is a need for analysing how and under what circumstances users and audiences engage with them. What factors influence how data visualisations are used, perceived or acted upon? This question, when asked of research and scientific evidence more generally, has a longer history going back to the work of Carol Weiss. Her concept of ‘research utilization’ opened up a field of enquiry seeking ‘to understand what “using research” actually means’ (Weiss, 1979: 426). Since then, many studies have shown how factors, including the perceived relevance of, and political polarisation around a topic contribute to research uses (Boswell, 2009; Contandriopoulos et al., 2010). Whether involving individuals, organisations or issue areas, ‘evidence is always contingent on context, sources, perceptions and timing’ (Levitt, 2013: 9).

Contingency makes research communication a complex phenomenon. It invites analysis of not only the objects and outputs that scientists produce but also how and in what contexts they do so (Brown, 2009; Suhay and Druckman, 2015). Studies into ‘knowledge brokerage’ which includes ‘all the activity that links decision makers with researchers’ (Lomas, 2007: 131) show how users connect with researchers via ‘intermediaries’ who translate and repackage information into more accessible forms. These intermediaries – which may include staff members of think tanks, news organisations or academic journals to name a few – use their roles as brokers to shape the knowledge flowing through them (Bandola-Gill and Lyall, 2017; Spiegelhalter, 2017).

This is also the case with data visualisations. For example, the creative director of *Nature* recently observed that the journal’s intended audience, along with organisational decisions about creative ownership, influences its visualisation practices (Krause, 2017). Alcíbar (2017) also emphasises the roles that graphical journalists played in visually communicating key aspects of the 2014–2016 Ebola epidemic in Spanish press outlets, arguing that they negotiate between norms of factual reporting and aesthetic attractiveness as they attempt to ‘make visible what is not’ (p. 1).

I aim to synthesise and develop these ideas by considering data visualisation as a mode of brokered research communication, echoing Frankel and Reid’s (2008) call for recognising how professional visualisers and science communicators contribute to visualisation outputs. I ask two questions: how do users outside of university settings engage with data visualisations and how do brokers contribute to their engagements? My argument is that visualisations are products of, as well as contributors to, knowledge brokerage processes. The processes of conceiving, creating, interpreting and responding to data visualisations as they occur within social, political and cultural contexts are what I call ‘visual brokerage’. Collectively, these brokers and processes generate a complex space for data visualisation that is characterised by mutual, bi-directional connections. Although data visualisation intends to make research more accessible and clear, it actually involves multiple stages and intermediaries. Consequently, they complicate how audiences and users make sense of visualisations as well as the information contained within them.

## 1. Knowledge brokerage and trust

Evidence and policymaking studies, as well as science and technology studies (STS), have revealed much about how researchers relate to the wider world. Scientists, as holder of particular kinds of expertise (Collins, 2014), engage with other individuals and organisations who use this expertise for a variety of purposes. These engagements are sometimes called ‘knowledge brokerage’ and comprise three categories: knowledge management, including finding, repackaging and sharing research; linkage and exchange that connect researchers with users or decision-makers; and capacity building that promotes stronger exchanges in the future (Ward et al., 2009).^1^ Early work on research brokerage identified how resources, such as knowledge and funding, flow in a bi-directional manner between academics and users through brokers (Sundquist, 1978). Brokers also create and add new kinds of knowledge in the process (Meyer, 2010). Diverse values, priorities and political contexts influence decisions taken by the groups involved (Oliver et al., 2014).

Brokerage also relates to the concept of ‘boundary objects’ developed in STS. Boundary objects are ‘scientific objects which both inhabit several intersecting social worlds … *and* satisfy the informational requirements of each of them’ (Star and Griesemer, 1989: 393). They are characterised by their ability to be mutually recognisable among different groups while adapting to specific needs in each of them. Political scientists use this concept as a way to study organisations, outputs or agreements that straddle academic science and public politics. These objects ‘facilitate collaboration between scientists and nonscientists, and they create the combined scientific and social order’ (Guston, 2001: 401). Furthermore, ‘they involve the participation of actors from both sides of the [politics-science] boundary, as well as professionals who serve a mediating role’ (Guston, 2001: 401). Knowledge brokers and boundary objects are similar, in that they translate and convey information among the intersecting worlds of research, politics and publics.

An important element of knowledge brokerage is credibility, whether directed towards research itself; the brokers who translate, transform or add to knowledge; or decision-makers who use and sometimes commission research. Credibility comprises perceptions about speakers’ trustworthiness – how open and honest they are – as well as their expertise about the topic at hand (Mackiewicz, 2010). Credibility can be specific to a particular moment or instance, such as a product review, or it can extend to other situations through one’s reputation.

## 2. Factors impacting engagement: Credibility and surprise

But how and under what circumstances does credibility influence how people engage with research? Two factors that are relevant for my argument, but by no means the only ones, include perceived credibility of the source and elicited emotions. Perceived credibility influences both what people think about an issue and how much importance they attach to it (Druckman, 2001). This happens because ‘citizens delegate to credible elites for guidance’ (Druckman, 2001: 1061). Instead of automatically taking on suggestions made by anyone, people evaluate how trustworthy and expert the source is. Known reputation is part of that evaluation.

Another factor relates to emotions elicited as people engage with information. Developed in psychology (Lerner and Keltner, 2000), this perspective argues that emotions mediate how people evaluate information by suggesting appropriate actions. Emotions ‘trigger a set of responses (physiology, behavior, experience, and communication) that enable the individual to deal quickly with encountered problems or opportunities’ (Lerner and Keltner, 2000: 476). These responses lead to actions or preferences that tend to fit with the triggered emotion. Previous research in political psychology and communication studies shows how emotions such as sympathy, anxiety and fear mediate whether certain kinds of frames have effects (Brader et al., 2008; Gross, 2008).

Among these, surprise is an ambiguous emotion. Psychologists debate whether it is positive, negative, mixed or neutral (Noordewier and Breugelmans, 2013). They do agree that surprise can lead people to be more aware of information at hand: it ‘is elicited by unexpected events, interrupting on-going thoughts and activities, and motivating people to pay attention to the unexpected stimulus’ (Noordewier and Breugelmans, 2013: 1327). Under certain circumstances, surprise leads to greater interest. There are two dimensions to surprise: novelty (‘how new is this information or experience?’) and coping potential (‘how able am I to make sense of this?’). If people experience something very new (having a high level of novelty) but feel unable to make sense of it (having a low level of coping potential), they are more likely to lose interest. As an emotion, surprise mediates how people engage with information. But its impact depends on self-evaluations of novelty and ability to cope with.

## 3. Data visualisation as a persuasive mode of communication

Since data visualisations are both particular objects of communication and processes involving researchers, brokers and audiences, it is important to ask what an ‘effective’ visualisation might look like and under what circumstances that effectiveness would arise. Some argue that effectiveness is about efficient use of space or design elements or saving users’ time (Chen et al., 2014; Kelleher and Wagener, 2011). A successful visualisation, according to these perspectives, enables users to quickly find desired information in reliable and easily understood ways.

Although this is an appealing definition of effectiveness, it has limitations. Many academic visualisations are made with other scientific experts in mind, rather than the general, non-expert public (Gough et al., 2014). Furthermore, as colleagues and I have argued elsewhere, viewers bring different priorities, skills and needs that influence how they interpret visual information (Kennedy et al., 2016b). Meanwhile, visualisations exist in social, political and cultural contexts just as other kinds of knowledge outputs and evidence do. They are communicative products in their own rights, intentionally and unintentionally privileging some values (e.g. scientific objectivity) over others, such as enjoyment or amusement (Kennedy et al., 2016a). Finally, viewers themselves hold perceptions about knowledge brokers and their outputs, ascribing levels of credibility to each.

Recent studies have begun to address these limitations by considering different audiences, emotions and contexts.^2^ Gough et al. (2014) advance the idea of ‘Visualisations for Non-Expert Users’ to link visualisation practitioners and audiences. They advocate for greater awareness of how people interpret design features, as well as the intentions of a visualisation. Meanwhile, Hullman and Diakopoulos (2011) identify how visualisations heighten the importance of some messages while diminishing others through ‘visual rhetoric’. Pandey et al. (2014) explore how visual representations of numbers influence perceptions, suggesting that this is due to an ‘increased sense of objectivity evidence supported by numbers carries’ (p. 2219)). Finally, Rall et al. (2016) explore how visualisations are used in human rights advocacy to persuade and convince.

These studies expand understanding about data visualisations in three important ways: (1) they place visualisations and their subsequent interpretation in social, cultural and political contexts; (2) they alert researchers to the ways that visualisation content and procedural decisions in creating them can influence viewers’ perceptions; and (3) they widen the category of ‘viewers’ beyond scientific experts to include people who may approach visualisations with different purposes and goals. The challenges that remain are to show how these understandings actually work in particular domains and with what implications. To address this problem, I share results from a study of how members of the British public reacted to three data visualisations containing data about migration in the United Kingdom.

## 4. Data and methods

There are several reasons why I focus on migration visualisations. First, migration is highly important for the British public and the United Kingdom’s political agenda (Blinder and Allen, 2016; Duffy and Frere-Smith, 2014), especially in the context of Brexit (Hobolt, 2016). It is a field in which many researchers have interests in communicating research with the British public, often through intermediaries such as civil society organisations (Allen, 2017b). Therefore, migration is a good proxy for other publicly relevant issues with substantial academic interest. Second, migration visualisations tend to contain features and presentation styles that appear in other scientific visualisations: for example, interactive maps and diagrams to indicate flows of people (Dennett, 2015). Finally, migration datasets are good examples of large, complex datasets that underpin other visualisations.

The data come from nine focus groups, one of which was a pilot, held between August and November 2014 in seven towns and cities around Great Britain. These locations were chosen on the basis of how rural or urban they were and the extent to which they had experienced increasing levels of immigration. The rationale for these dimensions was that people from regions with different experiences of migration might react differently to information about migration.^3^

Within these locations, the research design sought a wide range of people with different skills, backgrounds and levels of familiarity with either the visual (such as art) or data (such as computing and coding). This involved contacting organisations or community groups that brought together people who shared interests in sports, visual arts such as painting and sculpture, or data and computer programming. It also included migrants who had arrived in Britain at different times; mothers, professionals and volunteers in charitable organisations; and young farm workers.^4^ Altogether, 46 people agreed to participate. Focus groups ranged in size from two to six people. As a single-country, qualitative study, the research design did not intend to test hypotheses or causal mechanisms, leading to measurable changes in users’ opinions about migration.

**Figure 1.**
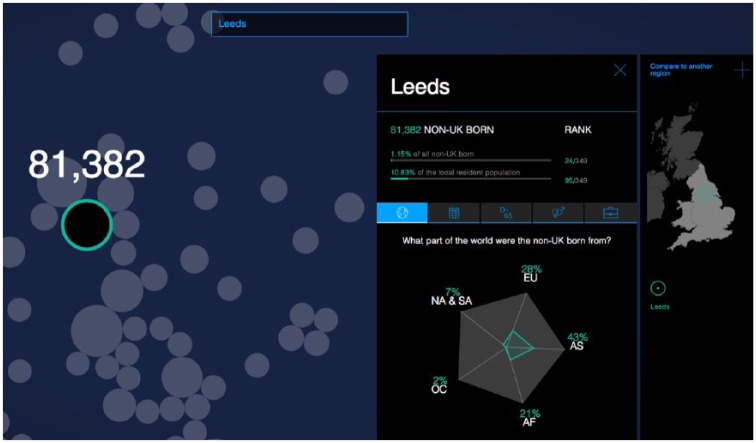
Screenshot of ‘migration in the census (V1-Census)’. Available at: http://www.compas.ox.ac.uk/migrationinthecensus.

Participants viewed and discussed up to eight visualisations, spanning a range of topics and styles that were chosen in consultation with a professional visualisation expert. In this article, I focus on the themes of credibility and surprise that emerged from analysing reactions to the three migration visualisations.^5^ ‘Migration in the Census’ (hereafter labelled V1-Census), seen in Figure 1, used census data from the Office for National Statistics (ONS) to show variation in the characteristics of foreign-born people in England and Wales. As an interactive visualisation, it allowed users to search and compare among local authority areas.

**Figure 2.**
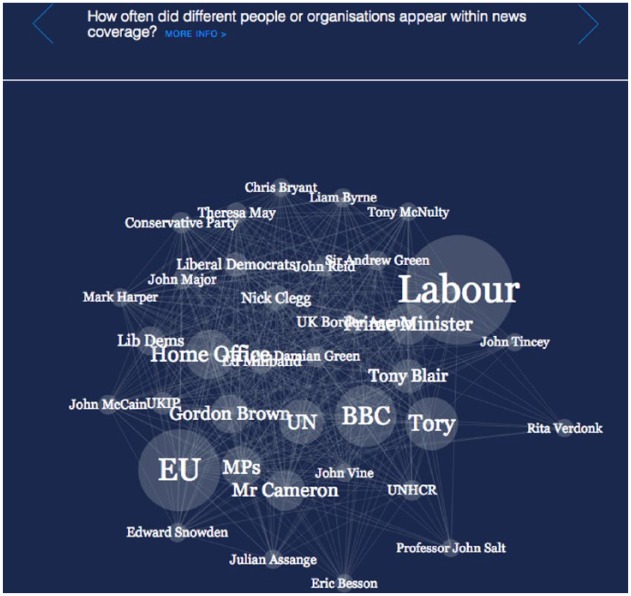
Screenshot of ‘Migration in the News (V2-News)’. Available at: http://www.compas.ox.ac.uk/migrationinthenews.

‘Migration in the News’ (V2-News), seen in Figure 2, was also an interactive visualisation. It visualised a quantitative analysis of how media outlets routinely described migrants, asylum seekers and refugees. Among other features, it allowed users to see which kinds of people or organisations appeared more frequently in news coverage, as well as how these differed between tabloid and broadsheet newspapers. Both V1-Census and V2-News were commissioned by The Migration Observatory at the University of Oxford and produced by an external design firm. Since I was associated with the Observatory at the time of the focus groups, I withheld my university and professional affiliations from participants until after the completion of the research to reduce social desirability bias in their responses.

**Figure 3.**
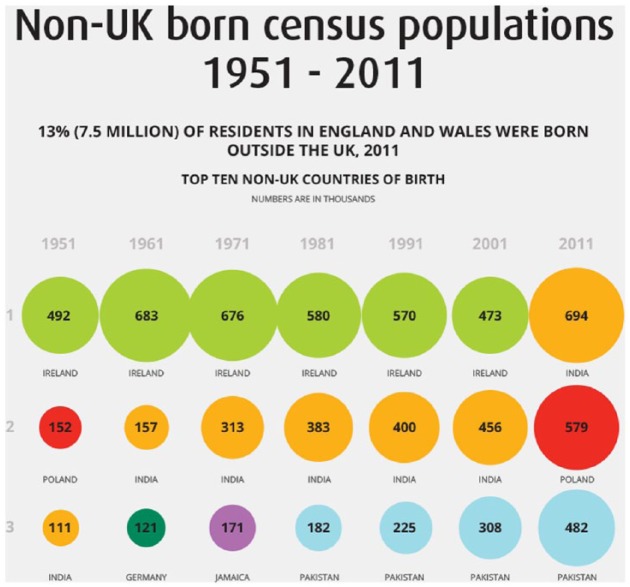
Excerpted screenshot of ‘Non-UK-Born Census Populations 1951–2011, Office for National Statistics (V3-Foreign Born)’. Available at: http://webarchive.nationalarchives.gov.uk/20160105222714/http://www.ons.gov.uk/ons/rel/census/2011-census-analysis/immigration-patterns-and-characteristics-of-non-uk-born-population-groups-in-england-and-wales/non-uk-born-census-populations-1951—2011—full-infographic.html.

Finally, ‘Non-UK Born Census Populations 1951-2011’ (V3-Foreign Born), seen in Figure 3, was a static output produced by the ONS. It displayed the top 10 countries of origin for non-UK-born residents in each census from 1951 to 2011, along with the share of the total foreign-born population that these 10 countries comprised. Importantly, all three migration visualisations used in this study were designed for non-academic members of the public to use for their own purposes.

Participants in each group looked at the set of visualisations on individual laptop computers or workstations. The visualisations were presented as standalone objects, accessed either as static images or as websites, rather than accompanied by news stories or other kinds of text. Participants had up to 30 minutes to look at, engage with and take notes on as many of the visualisations as they felt individually able. Then, the group members came together to discuss their reactions. Two researchers facilitated the discussion: one person tended to guide the discussion, while the other person redirected or rephrased questions to quieter participants (e.g. through prompts such as ‘what do you think?’) as well as welcomed divergent opinions (e.g. ‘Did other people find that the case, or not?’).

Focus groups were audio recorded and professionally transcribed. Then, responses to the visualisations were coded in NVivo, with particular attention given to the kinds of reasons that participants gave for their reactions towards each example. Participants agreed to share their broad occupations and education levels on a pre-participation questionnaire to provide context for their comments. Finally, to assure confidentiality when discussing responses, the participants chose their own pseudonyms that appear in the empirical material.

## 5. Empirical material

### Trust in brokers: Source credibility

The three visualisations about migration were explicitly branded as coming from reputable sources: The Migration Observatory in the case of V1-Census and V2-News, and the ONS in the case of V3-Foreign Born. Furthermore, both V1-Census and V3-Foreign Born heavily signalled that they were based on official 2011 UK Census data. Sally (48-year-old, British female with a degree) and Horace (28-year-old, British male working in the voluntary sector, educated to postgraduate level) commented in conversation with one another on the source reputation exhibited in V1-Census and V2-News:

Sally: Oxford University does give a certain authority. So, you would think if that data is on the Oxford University migration research centre, or whatever it’s called, then it is probably going to right.

Horace: I actually have the same bent on it in that saying it’s from Oxford University, I automatically assume it’s true.

Meanwhile, the two migration visualisations that relied upon census data produced reactions expressing trust. In these cases, participants’ knowledge and understanding about what censuses are – and their official character – came through in their responses:

Flora (68-year old, British female, retired, educated to A-level), explaining why she trusted V1-Census: *Well, because it is based on the census and I worked on the census some years ago so I know how the information is collected and I think that’s going to be pretty accurate.*

Sara (45-year old, British Pakistani female, part-time careers advisor, educated to postgraduate level): *The one that said the word ‘census’ [referring to V3-Foreign Born] I was more likely to believe because you think of official statistics … That’s why I liked it when I saw the word census, and thought ‘that looks official’*.

These reactions centred on the credibility of data collectors and sources – what they represented, the method of data collection and the relative believability of each. Although reputational aspects are rarely in the direct control of brokers or designers, especially when using secondary datasets, they nevertheless bear on how people perceive the resulting visualisation.

Users can also express distrust at a visualisation and – indirectly – the designers. As echoed in Rall et al. (2016), visual features help users to evaluate and make sense of the visualisation. For example, the perception of poor design can provoke disinterest, confusion or possibly doubt in the information. Robert (46-year-old, British male, business data analyst, educated to degree level) expressed frustration at both V1-Census and V2-News that were created by the same professional design company:

Robert: That census one [V1-Census] and the tabloid versus broadsheets’ one by the same company [V2-News], it was like designed by computers for computers and it was just bombarding you with things moving. Sometimes the things moved when you didn’t even have your hands on the mouse and it was just horrific. They probably had good intentions but I don’t know.

Participants also engaged with underlying research design choices as they evaluated whether to trust what they were seeing. Andrew (32-year-old, British male, working in food agriculture, educated to degree level) wondered why V2-News included a breakdown of how often various UK political parties (such as ‘Conservative’, ‘Labour’ and ‘UK Independence Party’) were mentioned. From the perspective of The Migration Observatory, who commissioned the visualisation, displaying this aspect seemed important for understanding which organisations the media mentions in relation to immigration. In other research focusing on the design process surrounding this specific visualisation, I show how decisions like these can stem from intermediaries’ own values and strategic objectives (Allen, 2017a; Kennedy and Allen, 2017). But for Andrew, the act of making these features especially visible was itself questionable:

Andrew: I don’t trust any of the ones with politics in it, like the census one had that bubble chart which showed you Labour and Tories and stuff.

Researcher: Are you talking about the media one?

Andrew: The migration one [V2-News]. And you could see you had the media part in the migration, yes, and it would tell you how many times they were mentioned in the past years.

Researcher: What didn’t make you trust it?

Andrew: I think any time it mentions that it just makes you wonder if it’s biased in any way, because why would it show you stuff about the different political groups? It makes you think. After the past couple of weeks anybody that makes something, whether it’s a TV programme or the news, have got some kind of link to politics in some kind of way. So, how do you know it’s not biased?

These responses suggest that users engage with the organisations and outputs as they determine how much they trust what they see. This finding, echoing prior research into how perceived source credibility matters for readers in text-based situations (Mackiewicz, 2010), is important for a fuller understanding of data visualisation as a mode of research communication. Credibility can come from the specific words used to describe a dataset in the moment of viewing, as Sara mentioned, or from previous experience such as in Flora’s case.

Situational factors also matter. Andrew’s suspicion about why V2-News would even attempt to show political information has to be interpreted in political, temporal and geographical contexts. That particular focus group occurred in Scotland during the period leading to the September 2014 referendum on Scottish independence. His perception is even more telling since V2-News does not contain any explicit reference to Scottish media. These findings open more questions about how and why certain people come to trust or distrust visualisations as forms of information.^6^

### Elicited emotions: The role of surprise

Another factor that scholars of political communication and psychology say influences how people engage with information is emotion (Van Kleef et al., 2015). According to these approaches, experiencing an emotion such as sadness, anxiety or contentment when encountering information makes it more likely that a person will subsequently react to the information in a way that fits with that emotion. Visualisations also present information in particular ways – some of which, intentionally or not, elicit emotions in viewers. These emotions can shape how people interpret and engage with the information and visualisation.

An emotion that regularly appeared across the focus groups was surprise. For example, V1-Census allowed users to select local authorities in England and Wales, and then see data about the characteristics of the non-UK-born population residing there as of March 2011 when the Census occurred. Many participants immediately looked for areas they were already familiar with, such as their hometown and current residence. Confronted with demographic data about the foreign-born population in these familiar areas, they expressed surprise when they compared what they saw with what they perceived:

Horace: But the actual data [in V1-Census] itself I was a little bit surprised at: it was either higher than I thought or it was lower than I thought. It kind of changed my perceptions of how I looked at it. For example, there were more South American migrants to East Northamptonshire than I thought there were.

Researcher: There were more?

Horace: Yes, there were more. Because I didn’t think it was a particularly high population, because it tends to be Poles, Lithuanian and Eastern European. But apparently there’s quite a high proportion of South Americans in East Northamptonshire; which on reflection is probably true, but that’s never occurred to me that I could be possibly biased subconsciously of how many people – possibly because it’s more noticeable.

Harriet: I thought it was interesting that on the migration one [V1-Census] I typed in places, I compared Chichester, which is where my sister lived for a little while, and she was like, ‘Oh it was very posh!’ and compared that in terms of the population to Bradford, where my friend went to university and was like, ‘There’s a lot of diversity here’, and saw that in percentages. And it was pretty well reflected to what I thought it would be, so yes, I learnt that.

Participants also were surprised by the information contained in V3-Foreign Born, produced by the ONS. One of the main patterns showed how most people living in the United Kingdom but born outside the country came from Ireland – and this held over six successive censuses from 1951 to 2001. For example, Jason (34-year-old, British male, working as a data scientist in information technology, educated to postgraduate level) had a strong reaction to this information:

Jason: Yeah. I was surprised that Irish immigrants were the most common in the UK. I think the last Census – it was the ONS [visualisation] again – it was surprising, it was something I hadn’t even thought of and it was like, ‘Wow!’. For all the talk of immigration things, the fact that Irish immigrants are the most common, for a lot of the time until ten years ago, it was something I didn’t expect. (Quoted in Kennedy and Hill, 2017: 7)

Besides information content, the local-scale feature designed into V1-Census also generated surprise. This was especially the case for participants who saw benefits for their professional work:

Theresa (50-year old, British female, works in local government, educated to A-level): It was like Christmas had come early for me, wow here’s all this information! Because there’s quite a few similar sort of data visualisations representing that type of information, but that was one that somehow I hadn’t seen before. So I did spend quite a lot of time playing with that. And I think it did because it had that personal and professional interest … It would highlight certain elements of labour market intelligence in local area, and I would then have to download the data. It would highlight certain aspects, but then I would try and get the more in-depth data.

The search function provided by V1-Census, as well as its interactive comparison function, seemed to facilitate this process – more than what might be expected from a traditional, tabular format.

Surprise interrupts a pre-existing activity or thought with something novel. Then, people evaluate their ability to handle this new information, which can lead to paying more attention to whatever caused the surprise in the first place (Noordewier and Breugelmans, 2013). This happened in several steps during the focus groups. First, visualisations enabled participants to pick out key trends, patterns and outliers: for example, the historical dominance of Irish migrants in V3-Foreign Born. This is already an important achievement in engaging with visualisation. Then, they compared this information to what they already felt they knew. When these comparisons did not match, it sometimes elicited surprise.

But surprise in itself does not necessarily predict a positive reaction. Tiffany (28-year-old, British female, working in local government as an advisor to businesses, educated to degree level with additional professional qualifications) also expressed surprise at V3-Foreign Born like Jason. However, after this initial emotional reaction prompted her to think more carefully and engage with the visualisation, she identified some annoying design flaws:

Tiffany: I think when I first looked at it [V3-Foreign Born] I was kind of like ‘oh!’ But when I really looked into it I thought actually it does make sense visually. You don’t actually need to do too much reading. What annoyed me was that the values were not clear. So, it said numbers are in thousands, in really small font, and I actually had to look for that. I thought they could have made that a wee bit clearer.

Of course, other factors in addition to emotions contribute to how people perceive information. Moreover, colleagues have identified how visualisations elicit a range of emotions besides surprise, too (Kennedy and Hill, 2017). But the focus groups revealed interesting evidence that showed how source credibility specifically interacts with surprise as people evaluate new information. For example, Sally linked these two factors as she explained her reaction to V1-Census: ‘even though I was quite surprised by some of the census figures, I did accept that they were true, mainly because it’s from a university’. References to the intermediary and sources of data contained within V1-Census appeared to influence how she engaged with the information contained within the visualisation despite its surprising and potentially challenging nature.^7^

## 6. Producing brokered spaces of data visualisation

I have aimed to apply knowledge brokerage theories to the mode of data visualisation, arguing that visualisations are both products and processes that connect researchers with users. But, as seen in critical debates around visualisation effectiveness, these connections’ shapes and forms are subject to the presence of brokers, as well as to social, cultural and political contexts.

**Figure 4.**
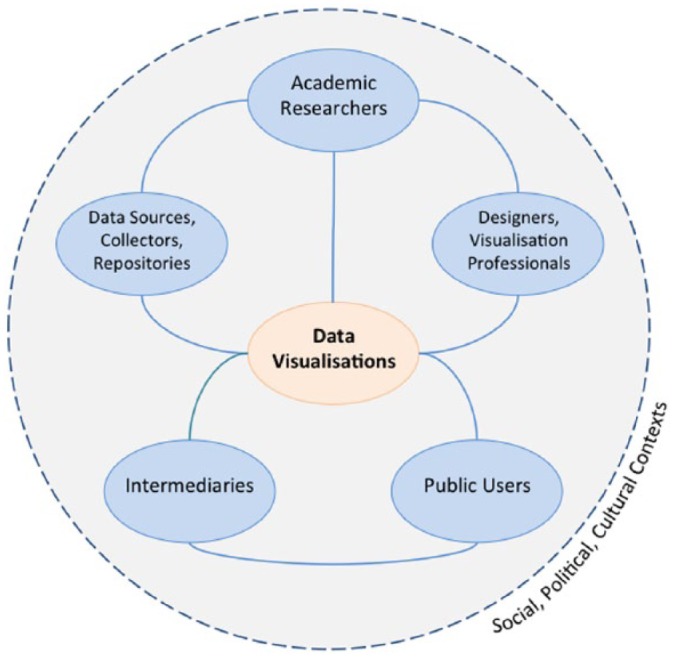
The visualisation space produced through visual brokerage.

Figure 4 depicts the brokered space produced by data visualisation.^8^ It arranges brokers that may operate in some, but not necessarily all, communication situations involving researchers, visualisations and users. At the heart of this space are data visualisations. All of components have boundary object statuses: they serve as potential points of contact between the worlds of research, politics and public debates. Furthermore, they exist within wider social, political and cultural contexts that are constantly changing.

‘Data collectors, sources, or repositories’ are people or organisations that find, aggregate or hold data. These might include survey companies, national statistical bureaus or news archiving services. ‘Designers or visualisation professionals’ are people and companies who engage with the creative and technical labour involved in making data visualisations. Designers, creative firms or freelancers who work for clients would fit this category. ‘Intermediary organisations’ liaise between researchers and users as they translate research, as well as possibly contribute their own knowledge. Examples include think tanks, civil society organisations such as charitable foundations, media organisations or journal editorial staff. In many cases, intermediaries play a large role in communicating visualisations to users: it is more likely that people encounter visualisations in their chosen media rather than directly accessing a statistical bureau website, for instance.

It is important to note that these categories are flexible and dynamic and may overlap in different circumstances. In one situation, individuals or organisations might be data collectors, while in another situation they may act as intermediaries. Or, researchers may take on more of a designer role when they conceive and sketch a visualisation that, in turn, becomes the brief that a professional visualiser fulfils.

Through different pathways indicated by solid lines, the illustration suggests multiple ways that academics link with members of the public, with varying degrees of engagement with brokers. Researchers may create their own visualisations based on data they collected by using freely available tools or existing software packages, and then distribute these through journal articles, blog posts or presentations. Or, they may rely on external companies to collect and initially analyse a dataset, on which visualisations may be based. If they do not have the necessary skills or time, researchers might enlist the help of designers to create more interactive and advanced visualisations. Researchers may also publish their visualisations with the help of intermediaries who have experience engaging with users in other arenas, including policy or media.

Mutual relationships also potentially exist among all of the boundary objects in the space. Users exert influence on researchers’ perceptions and practices through their responses to visualisations. People engaging with a visualisation could provide feedback that leads designers and researchers to consider other approaches or retain what resonated well. More likely, however, users can express preferences through different platforms (including social media or comments) for and against specific visualisations produced by intermediaries, who in turn may modify their own expectations and working practices with researchers as a result.

By extending the idea of brokerage to the visual realm, I argue that the barriers, facilitators and contextual features that are relevant to general knowledge brokerage also apply in the specific communication mode of data visualisation. Credibility, comprising trustworthiness and expertise, is an important factor that facilitates or constrains exchanges (Contandriopoulos et al., 2010). Through the concept of visual brokerage, I suggest that questions about credibility arise within this visualisation space at key links and nodes. Between data collectors and academic researchers, there might be questions about how the data were sampled and organised. The relationship between intermediaries and data collectors is also subject to criticism: why did these intermediaries choose a given source or topic in the first place? What motivates their choices? Similar questions arise as intermediaries or design professionals produce and engage with visualisations. Are there agendas or biases in the visualisation? Were the data and presentational choices selected to give particular impressions? Users also evaluate the credibility of data collectors, repositories and intermediaries: reputations, perceived quality of work and previous experience may shape reactions. Finally, recalling that visualisations do rhetorical and persuasive work (Kennedy et al., 2016a), users sometimes use the perceived quality and usefulness of visual designs as signals for believability. These factors are specific to visual modes of communication, rather than conventional text-based modes.

## 7. Conclusion

Researchers increasingly use data visualisations to communicate with users. Critical approaches to visualisations show how these objects affect perceptions through selective framing, while political communication theories suggest that emotions and perceptions of credible sources mediate these framings. By bringing these mechanisms together in the context of research communication, I have developed the concept of visual brokerage to advance two key arguments: visualisations are brokered products, and that processes of conceiving, creating, interpreting and responding to visualisations create a complex, brokered space that impacts how users engage with visualisations.

The empirical material revealed two key points about this dynamic visualisation space. First, perceived credibility of knowledge brokers (including data collectors, professional designers and intermediaries) influenced how people reacted to the information contained within the visualisations. Second, both the content within and design of the visualisations themselves elicited surprise, which also influenced how people engaged with the content. Jason’s surprise at finding out the numbers of Irish immigrants to the United Kingdom could have also come from a static table. But the uniquely visual and interactive elements elicited emotions and assessments of credibility. For example, as seen in Harriet’s use of V1-Census, the search function enabled her to quickly compare multiple places with which she was already familiar. This would probably have been more difficult to do using an equivalent list of migrants’ regional origins by local authority. Also, when Tiffany and Robert encountered design features they felt were flawed or inconvenient, they engaged with the visualisations differently.

This research suggests some implications for future studies and visualisation practice. First, researchers and brokers should recognise how visualisations exist in social, political and cultural contexts, which, in turn, may influence users’ engagement. Andrew’s scepticism expressed during the run-up to the Scottish independence referendum is a good example of this. Second, users bring prior feelings, assumptions or beliefs into the act of viewing. Future research could explore how individual-level factors such as attitudes, self-perceived numeracy and demographics contribute to users’ engagement. This would contribute to a better understanding of the extent to which persuasion occurs through visual means. Furthermore, it would be important to show if these effects, mediated by credibility and emotions, make an appreciable difference to outcomes such as public trust in science or perceptions of issues beyond migration. Although the study design did not aim to test for effects of data visualisation on changes on attitudes about migration, the concept of visual brokerage leaves this possibility open.

Third, researchers and intermediaries intending to use or create visualisations should consider how their audiences will perceive the data sources, intermediaries, research and aesthetic designs, and execution steps presented in – or indeed, left out of – the final product. Given the potential complexity present in many datasets and research projects, it is important to clearly state the definitions, concepts and procedures used. This might take the form of explanatory text with the visualisation that can deal with potential roadblocks to understanding, as well as be visually unobtrusive enough to facilitate an understanding of the main points.

Data visualisation is likely to grow in visibility and importance in science communication. As a form of brokered communication with users, it raises timely questions about scientists’ public engagement practices. When knowledge gained through research is used for many purposes and in new ways and public trust in expertise is arguably declining, these questions have urgent resonance. Bringing researchers, brokers and users into closer conversations about how information can be more effectively communicated through visual means seems like a vital step in response.

## Notes

1. Knowledge brokers’ duties are sometimes called ‘knowledge transfer (KT)’ or ‘knowledge exchange (KE)’ activities (Kitagawa & Lightowler, 2013).

2. Recent appreciation of how visualisations convey meaning has a longer history: see Monmonier (1996).

3. Previous studies suggest contact with migrants influences perceptions by either providing opportunities for friendships or heightening feelings of competition over resources such as jobs (McLaren, 2003).

4. Focus groups, when comprising people who share common features such as backgrounds, hobbies and interests, have practical as well as theoretical value for revealing how communication is both social and political: a group with similar features ‘establishes confidence more quickly, moves more readily beyond platitudes towards analysis, and still offers scope for exploring disagreements and contradictions’ (Lunt and Livingstone, 1996: 92).

5. Elsewhere, colleagues and I have also shared results relating to all of the visualisations (Kennedy and Hill, 2017; Kennedy et al., 2016a; Kennedy et al., 2016b).

6. Several studies do this for the subject of migration: see Sides and Citrin (2007) for a summary.

7. This relates to ideas of ‘motivated reasoning’ (Kunda, 1990) or ‘motivated scepticism’ (Taber and Lodge, 2006), where people are hypothesised to discount or reject factual information if it does not match their prior beliefs, especially on partisan issues. The evidence on this phenomenon, sometimes called the ‘backfire effect’ (Nyhan and Reifler, 2010), is still debated. In Sally’s case, it is possible that the relatively non-partisan topic (i.e. census results) and presentation mode (which avoided explicit editorialising) allowed other cues about the intermediary’s credibility to come through more strongly – although the research design cannot conclusively prove this.

8. I thank the anonymous reviewers for this point.

## References

[bibr1-0963662518756853] Alcíbar M (2017) Information visualisation as a resource for popularising the technical-biomedical aspects of the last Ebola virus epidemic: The case of the Spanish reference press. Public Understanding of Science 27(3):365–381. 28393588 10.1177/0963662517702047

[bibr2-0963662518756853] Allen W (2017a) Making corpus data visible: Visualising text with research intermediaries. Corpora 12(3): 459–482.

[bibr3-0963662518756853] Allen W (2017b) Factors that impact how civil society intermediaries perceive evidence. Evidence & Policy: A Journal of Research, Debate and Practice 13(2): 183–200.

[bibr4-0963662518756853] Bandola-Gill J Lyall C (2017) Knowledge brokers and policy advice in policy formulation. In: Howlett M Mukherjee I (eds) Handbook of Policy Formulation. Cheltenham: Edward Elgar Publishing, pp. 249–264.

[bibr5-0963662518756853] Blinder S Allen W (2016) UK public opinion toward immigration: Overall attitudes and level of concern. The Migration Observatory, 28 11 Available at: http://www.migrationobservatory.ox.ac.uk/resources/briefings/uk-public-opinion-toward-immigration-overall-attitudes-and-level-of-concern/ (accessed 29 February 2016).

[bibr6-0963662518756853] Boswell C (2009) The Political Uses of Expert Knowledge: Immigration Policy and Social Research. Cambridge: Cambridge University Press.

[bibr7-0963662518756853] Brader T Valentino NA Suhay E (2008) What triggers public opposition to immigration? Anxiety, group cues, and immigration threat. American Journal of Political Science 52(4): 959–978.

[bibr8-0963662518756853] Brown MB (2009) Science in Democracy: Expertise, Institutions, and Representation. Cambridge, MA: The MIT Press. 10.1007/s11024-011-9179-x PMC3167039 21957318

[bibr9-0963662518756853] Bucchi M Saracino B (2016) ‘Visual science literacy’: Images and public understanding of science in the digital age. Science Communication 38(6): 812–819.

[bibr10-0963662518756853] Chen M Floridi L Borgo R (2014) What is visualization really for? In: Floridi L Illari P (eds) The Philosophy of Information Quality. Berlin: Springer International Publishing, pp. 75–93.

[bibr11-0963662518756853] Collins H (2014) Are We All Scientific Experts Now? Cambridge: Polity Press.

[bibr12-0963662518756853] Contandriopoulos D Lemire M Denis J-L et al (2010) Knowledge exchange processes in organizations and policy arenas: A narrative systematic review of the literature. The Milbank Quarterly 88(4): 444–483. 21166865 10.1111/j.1468-0009.2010.00608.x PMC3037172

[bibr13-0963662518756853] Dennett A (2015) Visualising migration: Online tools for taking us beyond the static map. Migration Studies 3(1): 143–152.

[bibr14-0963662518756853] Druckman JN (2001) On the limits of framing effects: Who can frame? Journal of Politics 63(4): 1041–1066.

[bibr15-0963662518756853] Duffy B Frere-Smith T (2014) Perceptions and Reality: Public Attitudes to Immigration. London: Ipsos MORI.

[bibr16-0963662518756853] Frankel F (2002) Envisioning Science: The Design and Craft of the Science Image. Cambridge, MA: The MIT Press.

[bibr17-0963662518756853] Frankel F Reid R (2008) Big data: Distilling meaning from data. Nature 455(7209): 30–30.

[bibr18-0963662518756853] Gough P Ho X Dunn K et al (2014) Art and chartjunk: A guide for NEUVis. In: Nguyen QV Wu Y Bednarz T Huang T (eds) Proceedings of the 7th International Symposium on Visual Information Communication and Interaction Sydney, NSW, Australia: ACM, pp. 171–177.

[bibr19-0963662518756853] Gray E Jennings W Farrall S et al (2015) Small big data: Using multiple data-sets to explore unfolding social and economic change. Big Data & Society 2(1). Available at: http://bds.sagepub.com/content/2/1/2053951715589418.abstract

[bibr20-0963662518756853] Gross K (2008) Framing persuasive appeals: Episodic and thematic framing, emotional response, and policy opinion. Political Psychology 29(2): 169–192.

[bibr21-0963662518756853] Guston DH (2001) Boundary organizations in environmental policy and science: An introduction. Science, Technology, & Human Values 26(4): 399–408.

[bibr22-0963662518756853] Herring J VanDyke MS Cummins RG et al (2017) Communicating local climate risks online through an interactive data visualization. Environmental Communication 11(1): 90–105.

[bibr23-0963662518756853] Hobolt SB (2016) The Brexit vote: A divided nation, a divided continent. Journal of European Public Policy 23(9): 1259–1277.

[bibr24-0963662518756853] Hullman J Diakopoulos N (2011) Visualization rhetoric: Framing effects in narrative visualization. IEEE Transactions on Visualization and Computer Graphics 17(12): 2231–2240. 22034342 10.1109/TVCG.2011.255

[bibr25-0963662518756853] Kelleher C Wagener T (2011) Ten guidelines for effective data visualization in scientific publications. Environmental Modelling & Software 26(6): 822–827.

[bibr26-0963662518756853] Kennedy H Allen W (2017) Data visualisation as an emerging tool for online research. In: Fielding NG Lee RM Blank G (eds) The SAGE Handbook of Online Research Methods, 2nd edn. London: SAGE, pp. 307–326.

[bibr27-0963662518756853] Kennedy H Hill RL (2017) The feeling of numbers: Emotions in everyday engagements with data and their visualisation. Sociology 52(4):830–848.

[bibr28-0963662518756853] Kennedy H Hill RL Aiello G et al (2016a) The work that visualisation conventions do. Information, Communication & Society 19(6): 715–735.

[bibr29-0963662518756853] Kennedy H Hill RL Allen W et al (2016b) Engaging with (big) data visualizations: Factors that affect engagement and resulting new definitions of effectiveness. First Monday 21(11). Available at: http://firstmonday.org/ojs/index.php/fm/article/view/6389/5652

[bibr30-0963662518756853] Kitagawa F Lightowler C (2013) Knowledge exchange: A comparison of politics, strategies, and funding incentives in English and Scottish higher education. Research Evaluation 22(1): 1–14.

[bibr31-0963662518756853] Kirk A (2016) Data Visualisation: A Handbook for Data Driven Design. London: SAGE.

[bibr32-0963662518756853] Krause K (2017) A framework for visual communication at Nature. Public Understanding of Science 26(1): 15–24. 27117485 10.1177/0963662516640966

[bibr33-0963662518756853] Kunda Z (1990) The case for motivated reasoning. Psychological Bulletin 108(3): 480–498. 2270237 10.1037/0033-2909.108.3.480

[bibr34-0963662518756853] Lerner JS Keltner D (2000) Beyond valence: Toward a model of emotion-specific influences on judgement and choice. Cognition & Emotion 14(4): 473–493.

[bibr35-0963662518756853] Levitt R (2013) The Challenges of Evidence: Provocation Paper for the Alliance for Useful Evidence. London: NESTA Available at: https://kclpure.kcl.ac.uk/portal/files/14660122/Levitt_Nesta_Nov_2013.pdf (accessed 1 October 2015)

[bibr36-0963662518756853] Locke S (2011) Colouring in the ‘black-box’: Alternative renderings of scientific visualisations in two comic book cosmologies. Public Understanding of Science 22(3): 304–320. 23833056 10.1177/0963662511403877

[bibr37-0963662518756853] Lomas J (2007) The in-between world of knowledge brokering. British Medical Journal 334(7585): 129–132. 17235094 10.1136/bmj.39038.593380.AE PMC1779881

[bibr38-0963662518756853] Lunt P Livingstone S (1996) Rethinking the focus group in media and communications research. Journal of Communication 46(2): 79–98.

[bibr39-0963662518756853] Mackiewicz J (2010) The co-construction of credibility in online product reviews. Technical Communication Quarterly 19(4): 403–426.

[bibr40-0963662518756853] McLaren LM (2003) Anti-immigrant prejudice in Europe: Contact, threat perception, and preferences for the exclusion of migrants. Social Forces 81(3): 909–936.

[bibr41-0963662518756853] Meyer M (2010) The rise of the knowledge broker. Science Communication 32(1): 118–127.

[bibr42-0963662518756853] Monmonier MS (1996) How to Lie with Maps. Chicago, IL: University of Chicago Press.

[bibr43-0963662518756853] Noordewier MK Breugelmans SM (2013) On the valence of surprise. Cognition & Emotion 27(7): 1326–1334. 23560688 10.1080/02699931.2013.777660

[bibr44-0963662518756853] Nyhan B Reifler J (2010) When corrections fail: The persistence of political misperceptions. Political Behavior 32(2): 303–330.

[bibr45-0963662518756853] Oliver K Innvar S Lorenc T et al (2014) A systematic review of barriers to and facilitators of the use of evidence by policymakers. BMC Health Services Research 14(2): 2. 24383766 10.1186/1472-6963-14-2 PMC3909454

[bibr46-0963662518756853] Pandey AV Manivannan A Nov O et al (2014) The persuasive power of data visualization. IEEE Transactions on Visualization and Computer Graphics 20(12): 2211–2220. 26356935 10.1109/TVCG.2014.2346419

[bibr47-0963662518756853] Rall K Satterthwaite ML Pandey AV et al (2016) Data visualization for human rights advocacy. Journal of Human Rights Practice 8(2): 171–197.

[bibr48-0963662518756853] Sides J Citrin J (2007) European opinion about immigration: The role of identities, interests and information. British Journal of Political Science 37(3): 477–504.

[bibr49-0963662518756853] Spiegelhalter D (2017) Trust in numbers. Journal of the Royal Statistical Society: Series A (Statistics in Society) 180: 948–965.

[bibr50-0963662518756853] Star SL Griesemer JR (1989) Institutional ecology, ‘translations’ and boundary objects: Amateurs and professionals in Berkeley’s Museum of Vertebrate Zoology, 1907-39. Social Studies of Science 19(3): 387–420.

[bibr51-0963662518756853] Suhay E Druckman JN (2015) The politics of science: Political values and the production, communication, and reception of scientific knowledge. The ANNALS of the American Academy of Political and Social Science 658(1): 6–15.

[bibr52-0963662518756853] Sundquist JL (1978) Research brokerage: The weak link. In: Lynn LE (ed.) Knowledge and Policy: The Uncertain Connection (Study Project on Social Research and Development). Washington, DC: National Academy of Sciences, pp. 126–144.

[bibr53-0963662518756853] Taber CS Lodge M (2006) Motivated skepticism in the evaluation of political beliefs. American Journal of Political Science 50(3): 755–769.

[bibr54-0963662518756853] Tufte ER (1983) The Visual Display of Quantitative Information. Cheshire, CT: Graphics Press.

[bibr55-0963662518756853] Van Kleef GA van den Berg H Heerdink MW (2015) The persuasive power of emotions: Effects of emotional expressions on attitude formation and change. Journal of Applied Psychology 100(4): 1124–1142. 25402955 10.1037/apl0000003

[bibr56-0963662518756853] Ward V House A Hamer S (2009) Knowledge brokering: The missing link in the evidence to action chain? Evidence & Policy: A Journal of Research, Debate and Practice 5(3): 267–279. 10.1332/174426409X463811 PMC3024540 21258626

[bibr57-0963662518756853] Weiss CH (1979) The many meanings of research utilization. Public Administration Review 39(5): 426–431.

[bibr58-0963662518756853] Welles B Meirelles I (2015) Visualizing computational social science. Science Communication 37(1): 34–58.

